# The longitudinal effects of STEM identity and gender on flourishing and achievement in college physics

**DOI:** 10.1186/s40594-018-0137-0

**Published:** 2018-11-30

**Authors:** Viviane Seyranian, Alex Madva, Nicole Duong, Nina Abramzon, Yoi Tibbetts, Judith M Harackiewicz

**Affiliations:** 1 Psychology Department, California State Polytechnic University, Pomona, 3801 West Temple Avenue, Pomona, CA 91768 USA; 2 Division of Behavioral and Organizational Sciences, Claremont Graduate University, 123 E. 8th Street, Claremont, CA 91711 USA; 3 Curry School of Education, University of Virginia, Ruffner Hall RM 287 405 Emmet St., Charlottesville, VA 22904 USA; 4 Department of Psychology, Brogden Hall, University of Wisconsin, Madison, 1202 West Johnson Street, Madison, WI 53706-1611 USA

**Keywords:** STEM, STEM identity, Young women and STEM, Social identity theory, Physics education, Flourishing, Force concept inventory, Well-being, Positive psychology, Positive education

## Abstract

**Background:**

Drawing on social identity theory and positive psychology, this study investigated women’s responses to the social environment of physics classrooms. It also investigated STEM identity and gender disparities on academic achievement and flourishing in an undergraduate introductory physics course for STEM majors. One hundred sixty undergraduate students enrolled in an introductory physics course were administered a baseline survey with self-report measures on course belonging, physics identification, flourishing, and demographics at the beginning of the course and a post-survey at the end of the academic term. Students also completed force concept inventories, and physics course grades were obtained from the registrar.

**Results:**

Women reported less course belonging and less physics identification than men. Physics identification and grades evidenced a longitudinal bidirectional relationship for all students (regardless of gender) such that when controlling for baseline physics knowledge: (a) students with higher physics identification were more likely to earn higher grades; and (b) students with higher grades evidenced more physics identification at the end of the term. Men scored higher on the force concept inventory than women; although no gender disparities emerged for course grades. For women, higher physics (versus lower) identification was associated with more positive changes in flourishing over the course of the term. High-identifying men showed the opposite pattern: negative change in flourishing was more strongly associated with high identifiers than low identifiers.

**Conclusions:**

Overall, this study underlines gender disparities in physics both in terms of belonging and physics knowledge. It suggests that strong STEM identity may be associated with academic performance and flourishing in undergraduate physics courses at the end of the term, particularly for women. A number of avenues for future research are discussed.

A mental health crisis is evident among college students today (American Psychology Association, 2018). The American College Health Association–National College Health Assessment (2015) reports that 53% of students in their survey reported feeling hopeless and 39% reported feeling so depressed that they found it difficult to function within the last 12 months. University counseling centers, faculty, staff, friends, and parents may be critical sources of support for university students around mental health and illness on campus, yet it is important to ask what should universities and colleges do to create social environments where students can thrive not only in terms of academics but also psychologically? In other words, how can all students from all walks of life develop and maintain strong academic performance as well as positive psychological health through positive and inclusive university experiences, in which students capitalize on their strengths to actualize meaningful lives and careers (Schreiner et al. 2009)? This question is critical for education. We propose that answering this question requires the alignment of educational psychology with social and positive psychology to study *both* cognitive academic performance *and* psychological well-being as outcomes of educational interventions and endeavors (Adler, 2017)—an idea termed *positive education* (Seligman et al. 2009). The current study applies a positive education perspective to focus specifically on the experiences of university women in physics and how they differ from those of men. We also explore the potentially powerful role that social identification (Tajfel and Turner, 1986) may play in both academic performance and flourishing.

## Women in STEM

Women trail behind men in their numerical representation in Science, Technology, Engineering, and Mathematics (STEM) fields (National Science Foundation, 2015). For example, only 1 of 5 physicists are women (Ivie and Guo, 2006) and only 8% of full professors in physics are women (Ivie et al. 2013; Abramzon et al. 2015). Senior-level physics faculty positions are still predominantly held by men. The underrepresentation of women is particularly evident in undergraduate university STEM classrooms and is pronounced in physics. For example, fewer women than men major in STEM and less than 20% of Bachelor’s degrees in physics are awarded to women (IPEDS survey, American Physics Society, 2015). Additionally, more women switch out of STEM majors than men (Seymour and Hewitt, 1997). This striking gender disparity creates a social context in STEM academic environments that signals to women that they are numerical minorities who may not belong in the field (Murphy et al. 2007), thereby potentially reducing interest in pursuing STEM fields and occupations (Kim et al. 2018; Master et al. 2016; Stout et al. 2011; Walton and Cohen, 2007). Underrepresentation is not the only signal that women do not belong. Women also report overt sex discrimination in STEM majors (Steele et al. 2002) and occupations (Funk and Parker, 2018). For example, 80% of a sample of 1350 female physicists representing 70 countries across the world report that attitudes about women in physics needs to be improved (Ivie and Guo, 2006). Sixty percent of these same female physicists also report that the level of gender discrimination in STEM needs improvement. Additionally, a recent survey of women in physics shows that women accumulate fewer resources and have fewer opportunities in physics than men (Ivie and White, 2015).

How can more women be encouraged to pursue degrees in STEM fields? To address this question, the current study first examines responses to the social climate of undergraduate physics courses. Specifically, we examine gender differences in course belonging. If gender differences in belonging exist, it may signal that women feel relatively marginalized inside the classroom. Next, we examine the student experience within higher education by examining ways that students can reach higher levels of both flourishing and academic performance—a goal of positive education. Specifically, we explore whether STEM identification is related to better academic performance and flourishing for students in STEM, particularly for women. If this relationship is positive, then future research examining STEM identification may be warranted as a potential intervention route for women in STEM.

## The social environment of physics classrooms

Women in STEM may respond in various ways to social environments that signal (directly or indirectly) that they are underrepresented minorities. First, women may experience lower levels of social belonging within the environment due to their female social identities. Belonging is a fundamental need (Baumeister and Leary, 1995), and environments that do not meet fundamental belonging needs may be less attractive to individuals. Since higher education requires a considerable investment of time and energy (4–6 years), being in a social environment that meets one’s belonging needs may be particularly important for persistence. Navigating a social environment for extended periods of time where one does not feel that one belongs can be taxing and aversive. Indeed, research shows that individuals from underrepresented groups are less likely to feel that they belong (Rainey et al. 2018). Lower social belonging in academic settings is stressful (Townsend et al. 2011; Grobecker, 2016) and affects interest in a field (Cheryan et al. 2009; Murphy et al. 2007), academic performance (Murphy and Zirkel, 2015), and well-being (van Laar et al. 2010). In the current study, our first research question addressed women’s reactions to the social environment of physics courses. Did women experience less social belonging in physics courses than men? (research question 1) To address this question, the self-reported levels of belonging of women in introductory physics courses were compared to those of men. Students also rated their levels of belonging to the *university* as a whole, which allowed us to tease apart whether potential gender differences in social belonging levels were course/field-specific or university-wide. Given that physics is a male-dominated field, it is hypothesized that women will report less belonging in the courses than men (hypothesis 1) and no significant gender differences would emerge for belonging to the university.

## Towards positive higher education: Student flourishing as a new educational goal

A key question is how women in male-dominated STEM fields such as physics can have their belonging needs met and achieve strong academic performance and positive psychological health through positive and inclusive university experiences where students successfully actualize meaningful lives and careers. We propose that a meaningful step in this direction is to operationalize students’ well-being through a new conceptual framework called *flourishing*—a concept from positive psychology that taps into psychological health (Seligman, 2011; Fredrickson and Losada, 2005; Diener et al. 2010). Individuals flourish when they “live within an optimal range of human functioning, one that connotes goodness, generativity, growth, and resilience” (Fredrickson and Losada, 2005, p. 678). Flourishing is a gauge of well-being that goes beyond traditional measures of well-being that tend to focus on depression or anxiety or positive states such as life satisfaction, happiness, or positive emotion (Huppert and So, 2013). Seligman (2011) defines flourishing in terms of the presence of five elements (abbreviated as *PERMA*): (1) the presence of *positive emotion* such as happiness and life satisfaction; (2) *engagement* in activities and tasks that help us reach a state of flow (Nakamura and Csikszentmihalyi, 2009); (3) positive *relationships* and social connections; (4) living a life with *meaning* (subjectively defined); and (5) the *accomplishment* of goals for their own sake.

Flourishing is aligned with the goals of higher education in that universities are no longer charged with just graduating people with degrees, but with graduating individuals who are critical thinkers and life-long learners who seek to grow and make positive and innovative contributions to twenty-first century society (see Schreiner et al. 2009). Put simply, higher education should provide environments, opportunities, and scaffolding for students to flourish. We propose that the development of a strong STEM identity may be one way that women may flourish and achieve strong academic performance in STEM fields. This is because the development of STEM identity, particularly from a social identity perspective, directly implicates social connections, relationships, and the social self—an important element of flourishing.

## A social identity perspective on STEM identity, gender, and achievement

A considerable body of work examines STEM identity among women and other underrepresented minority (see Kim et al. 2018, for a thorough review of the literature on STEM identity for adolescent women). For instance, research on undergraduates, graduate students, and postdoctoral scholars shows that an identity as a scientist predicts commitment to a science career (Chemers et al. 2011; Robnett, 2012). Research employing cluster analysis also shows that self-doubting women who are high achievers are also low in identity as a scientist (Robnett and Thoman, 2017). Although various researchers define a STEM identity in terms of an *identity as a scientist* (occupational identity), we define STEM identity as a type of *social identity* in line with Kim and colleagues (2018). This is an important distinction as viewing a STEM identity through a social identity perspective implicates the social self. A social identity (Tajfel and Turner, 1986) comprises individuals’ group membership (e.g., physicist) and the extent to which people identify with a group and see themselves as a group member (e.g., physics major). Social identities provide important information about the meaning of group membership in two ways. First, they outline the boundaries of group membership—who belongs to a group (e.g., physicists) and who does not (e.g., sociologists). Second, social identities describe what it means to be a group member by detailing descriptions of the content (or prototypes) of social identity (Hogg and Abrams, 2001). Social identity content comprises *ingroup prototypes* (Turner, 1991), which are fuzzy sets of group-based attributes—norms, attitudes, traits, values, behaviors, and stereotypes—that define a typical group member and distinguish her/him from other groups (Hogg, 2007). Individuals who embody the ingroup prototype—that is, who act as representative and typical group members—tend to occupy central positions of influence (e.g., leaders) within groups (Hogg, 2001).

From a social identity perspective, a STEM identity is a type of social identity concerning the extent to which individuals identify as members of a specific STEM field (e.g., physics major, physicist) and see themselves and others in terms of specific prototypes of the STEM field (e.g., “physicists are nerds”) (Kim et al. 2018). Applied to undergraduate students, students with strong STEM identities likely define themselves in terms of their specific “STEM major” (e.g., physics) and identify with their field. Research consistently shows that individuals who highly identify with a social identity are more likely to be influenced by ingroup prototypes and to strive to align their own attitudes and behaviors in line with them (Ellemers et al. 2002; Hogg, 2007). Therefore, high STEM identifiers are more likely than low identifiers to strive to conform to the prototypes of the field as embodied in and espoused by professors and peers in their major.

## Do men identify with physics and academically perform better than women?

Given that physics is a male-dominated STEM field and physics prototypes may be more closely aligned with men than women, men may be more aligned with physics prototypes, making them more prone to identify with physics than women. For example, content analyses of scientific advertising revealed more images of men than women (Barbercheck, 2001), and successful scientists are seen as more similar in personality to men than women (Carli et al. 2016). With male physics prototypes likely abounding in physics, women may feel like outgroup members and outsiders, thereby reducing identification with the field and the motivation to excel academically in the field and pursue the field. This may also help to explain consistent findings that women score lower on the FCI than men (e.g., Docktor and Heller, 2008; McCullough, 2011; Traxler et al. 2018). This reasoning leads to various research questions and hypotheses: (1) are there gender differences in physics identification before and after students take an introductory physics course? (research question 2). It is hypothesized that men will identify more with the field of physics than women both before and after completing an introductory physics course (hypothesis 2); (2) are there gender differences in FCI scores and physics grades (research question 3)? It is hypothesized that men will earn higher course grades (hypothesis 3a) and acquire more knowledge (hypothesis 3b) in the physics course than women.

## Does STEM identity increase academic achievement?

Given that STEM prototypes emphasize intelligence and dedication to science (Kim et al. 2018) and are likely to be linked with high academic achievement and innovation, higher STEM identification may be associated with higher academic achievement. In line with this prediction, two studies conducted on university samples in Europe show that higher student identification with their discipline predicted deeper learning, more positive ratings of their learning communities, and higher grades (Bliuc et al. 2011). Moreover, a study at an Australian university showed that discipline-related social identity predicted learning approaches, and that perceived norms prevalent in the learning environment (prototypes) moderated this effect (Platow et al. 2013). It is important to note that research conducted by (Platow et al. 2013) on undergraduate psychology students in Australia, however, did not show that discipline social identity predicted deep learning over time. This study evidenced the opposite pattern—that deep learning predicts discipline social identification. In this way, learning discipline-related content affected the way that individuals viewed themselves and the extent to which they perceive that they share common norms, attitudes, and values with the discipline.

Overall, the direction of the relationship between social identification and knowledge and performance remains unclear. To shed light on this issue, the next set of research questions asked: (a) to what extent does physics identification at the beginning of the term in an introductory physics course predict various achievement outcomes (outlined below) at the end of the term? (research question 4); (b) To what extent does academic achievement predict STEM identification at the end of the term? (research question 5). Drawing on previous research, the following hypotheses were tested: controlling for pre-term physics knowledge (FCI scores), higher physics identification at the end of the term will be related to higher scores on a physics knowledge test (FCI scores) (hypothesis 4a) and higher physics course grades (hypothesis 4b) at the end of the term. Additionally, controlling for pre-term physics knowledge (FCI scores), higher post physics knowledge scores (hypothesis 5a) and physics course grades (hypothesis 5b) will be related to more physics identification at the end of the term.^1^

## Does STEM identity increase flourishing?

**Fig. 1:**
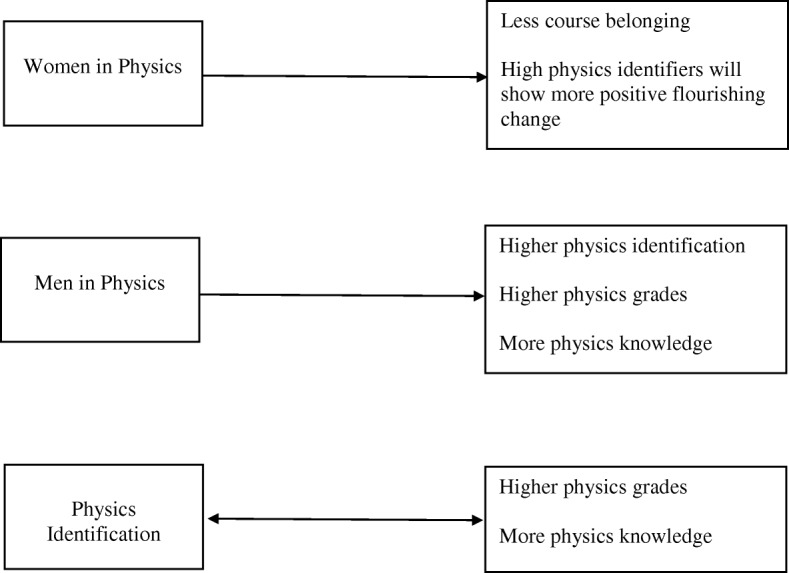
Hypothesized relationships

A robust body of research underlines the profound mental and physical benefits of social connection that are associated with valued social identities (Greenaway et al. 2016). These benefits hold true for social identifications in educational settings (Bizumic et al. 2009; Cameron, 1999) and even for the acquisition of new group memberships. The more groups one joins and identifies with, the more benefits are accrued. For instance, the risk of depression relapse is reduced by 64% by joining three groups that one highly identifies with versus 24% by joining one group (Cruwys et al. 2013). Applied to STEM identities in higher education, students who are studying a discipline and learning about a STEM field may be in the process of “trying on” and acquiring new STEM identities. This may be particularly true for women, who are underrepresented in some STEM fields and may be less likely to identify with physics than men (see H4 above). For women in particular, identifying with physics may provide a new sense of “who I am” and how one fits into the social environment, which may not only reduce depression, as per Cruwys and colleagues (2013), but it may also increase flourishing over time. This is because increasing identification with STEM may help to meet women’s global needs (Greenaway et al., 2016) and reduce a sense of self-uncertainty (Hogg, 2007) by providing a prescription of “who to be” and a pathway towards potential careers. It may provide women with a new social self—new social connections, new ingroups with peers, and new leaders, and role models. This may prove beneficial particularly at the beginning of students’ university trajectories, when students may experience stressors associated with transitioning to a new university setting and potentially new living situations. This leads to a final research question: does STEM identification predict gains in student flourishing over time, particularly for women? (research question 6). To address this research question, we examined the relationship of gender and physics identification on gains in flourishing over the course of a term while students were enrolled in an introductory physics course. It was hypothesized that students who highly identify with physics would show gains in flourishing compared to low identifiers and that this relationship may be moderated by gender such that the relationship would be most pronounced for women (hypothesis 6). See Table 1 for a list of research questions and hypotheses. Figure 1 depicts hypothesized relationships between variables.

**Table 1:** Research questions and hypotheses

Research questions	Hypotheses
1. Did women experience less social belonging and more belonging uncertainty in physics courses than men?	1. Women will report less belonging in the course than men.
	(No gender differences will emerge for belonging at the university level)
2. Are there gender differences in physics identification before and after students take an introductory physics course?	2. Men will identify more with the field of physics than women both before and after completing an introductory physics course.
3. Are there gender differences in FCI scores and physics grades?	3a. Men will earn higher course grades than women.
	3b. Men will acquire more knowledge in the physics course than women.
4. To what extent does physics identification predict various achievement outcomes at the end of the term?	4a. Controlling for pre-term physics knowledge (FCI scores), higher physics identification at the end of the term will be related to more physics knowledge.
	4b. Controlling for pre-term physics knowledge (FCI scores), higher physics identification at the end of the term will be related to higher physics course grades.
5. To what extent does academic achievement predict STEM identification at the end of the term?	5a. Controlling for pre-term physics knowledge (FCI scores), higher physics knowledge scores will predict higher physics identification at the end of the term.
	5b. Controlling for pre-term physics knowledge (FCI scores), higher physics grades will predict higher physics identification at the end of the term.
6. Does STEM identification predict gains in student flourishing over time, particularly for women?	6. Students who highly identify with physics show gains in flourishing compared to low identifiers, and this relationship may be moderated by gender such that the relationships would be most pronounced for women.

## The current research

The current study had three goals: (1) to examine gender differences social belonging in the physics classrooms; (2) to examine gender differences in physics identification and physics academic achievement; and (3) to assess the relationship of physics identification and gender on academic achievement and flourishing change. This longitudinal study was conducted in an introductory physics course for STEM majors (except life science majors). During required laboratory sections, students who agreed to participate in the study completed surveys on “how to improve physics education” at the beginning of the term and at the end of the term along with physics knowledge assessments. Participants provided consent for the research team to access their course grades. Overall, it was predicted that gender differences would exist in course belonging, physics identification, and academic achievement. It was also predicted that physics identification would affect achievement outcomes and that the influence of physics identification on flourishing change would be moderated by gender.

## Method

### Participants

Undergraduate students (*N* = 160) enrolled in an introductory physics course for STEM majors (except life science majors) and a co-requisite lab at a university in the Western United States were recruited for the study. Participants were sampled from three sections of the same course; two sections (*n* = 104; *n* = 100) were taught by a female professor and one section was taught by a male professor (*n* = 63). Participants in the sample consisted of the following STEM majors: engineering (67.4%), computer science/computer information systems (17.5%), mathematics (7.5%), biochemistry/biotechnology/chemistry (6.3%), geology (.6%), and physics (.6%). Males constituted the majority of students (72.5%) and females were in the minority (27.5%). The student sample was ethnically diverse: 47.5% White/Caucasian, 39.4% Hispanic/Latino, 20% Asian/Asian American, 17.5% did not indicate their ethnicity, 6.2% consisted of 2 or more races, 4.4% African American/Black/African, 3.8% Pacific Islander, and .6% Native American/Alaska Native. Participant ages ranged between 18 and 34 years (*M*_age_ = 19.38, SD = 2.56), with 76.3% of participants in their first year of college, 6.9% second year, 9.4% third year, 2.5% fourth year, and 5.1% indicated being in their fifth year or beyond or did not indicate their year in college (refer to Table 2 for demographic characteristics). The gender and ethnic makeup of each physics class was comparable to the whole sample.

**Table 2:** Characteristics of the physics students sample

Demographic characteristics	%
STEM major	
Engineering	67.4
Computer Science/Computer Information Systems	17.5
Mathematics	7.5
Biochemistry/Biotechnology/Chemistry	6.3
Geology	.6
Physics	.6
Gender	
Males	72.5
Females	27.5
Ethnicity	
White/Caucasian	47.5
Hispanic/Latino	39.4
Asian/Asian American	20
African American/Black/African	4.4
Pacific Islander	3.8
Native American/Alaska Native	.6
Did not indicate ethnicity	17.5
Consisted of two or more races	6.2
Year in college	
First year	76.3
Second year	6.9
Third year	9.4
Fourth year	2.5
Fifth year or beyond or did not indicate	5.1

*N* = 160. Participants’ ages ranged from 18 to 34 years (*M*_age_ = 19.38, *SD* = 2.56)

### Procedure

The first survey was distributed at the beginning of the quarter (week 1) in 12 different sections of physics labs by the research team (three of the authors and research assistants). Approximately 15 min after a lab started, a research team member recruited participants by asking students to participate in a study designed to “improve physics education” and emphasized the confidentiality of participant responses. Participants who elected not to participate were asked to do a quiet activity for the duration of data collection. Participants took approximately 10–15 min to complete the survey. Once consent forms and surveys were completed, they were collected by the research team and participants were thanked for their time. Close to the end of the quarter (week 9), participants once again completed surveys during lab sessions. One lab section did not complete the second survey because the lab session was canceled. Most students in the physics labs and in our sample volunteered to complete both surveys (88.8%). The female professor’s two course sections had a response rate of 54.8% and 70% respectively, of students who completed both surveys. The male professor’s course section had a response rate of 73% of students who completed both surveys.

### Measures

#### Physics identification

Two items tapped into *physics identification* (“I identify with the field of physics,” “the field of physics is a good fit for me”) on a scale a scale ranging from 1 (not at all true) to 7 (very true) (α = .74). Physics identification was measured at the beginning and end of the term. The beginning of the term measure was employed in all analyses.

#### Social belonging

Two measures of social belonging were employed on response scales ranging from 1 (not at all true) to 7 (very true). *Course belonging* consisted of two items that asked participants to rate: (a) I am not sure if I belong in this course, and (b) I belong in this course (α = .65). *Belonging to the university* included four items: (a) I belong at this university, (b) I feel like this university is a good fit for me, (c) I feel welcome on this campus, and (d) I am satisfied with my experience at university (α = .87).

#### The force concept inventory

The *force concept inventory* (Hestenes et al. 1992) is a 29-item physics knowledge diagnostic tool. It is designed to test the Newtonian concept of force, which is essential to the understanding of mechanics. It taps into the extent to which students hold misconceptions in physics. The inventory may be used to assess whether students who have taken an introductory physics course are ready to move on to more advanced physics courses. The FCI was administered by faculty in the university’s physics and astronomy department to the same students as the current sample at the beginning and the end of the same term while the study was in progress. The post measure is considered most meaningful and was employed in all analyses.

#### Flourishing

Due to survey space constraints, we employed an 8-item measure of flourishing (see Appendix) called the *Flourishing Scale* (FS) (α = .89) (Diener et al. 2010), which aligns with Seligman’s (2011) five pillars of flourishing. Note that a longer 23-item measure of flourishing called the PERMA profiler also exists (Butler and Kern, 2016). All items were answered using a scale ranging from 1 (strongly disagree) to 7 (strongly agree). Flourishing was measured at the beginning of the term (baseline) and at the end of the term (post). Difference scores in flourishing were calculated by subtracting end of the term flourishing scores from the beginning of the term scores. Positive numbers indicated positive change in flourishing (increased student well-being over time) and negative numbers indicate negative change in flourishing (worse student well-being over time).

#### Physics course grades

Physics course grades were obtained from the registrar’s office. The research team asked students permission to access their end-of-term physics course grades and most participants agreed (*n* = 159). Physics course grade was measured on a scale ranging from 1 (*fail*) to 4 (A—*excellent*), which is in line with widespread grade point average calculations in the United States.

#### Demographics

Demographic data collected included age, ethnic/racial background, gender, major, and year in college.

## Results

Intercorrelations and descriptive statistics for all continuous variables are listed in Table 3. Note that for all analyses with independent samples *t* tests, only relevant statistics for equal variances not assumed were reported due to uneven samples size for analyses (women *n* = 44 and men *n* = 116).

**Table 3:** Mean scores, standard deviations, and intercorrelations for all continuous variables

Variables	*M*	SD	1	2	3	4	5	6	7
1. Course belonging post	5.43	1.32							
2. University belonging post	5.55	1.16	.39***						
3. FCI baseline	12.52	6.06	.28***	.08					
4. FCI post	15.90	6.38	.23*	.078	.81***				
5. Physics grade	2.91	1.01	.23**	.10	.39***	.32**			
6. Flourishing change	−.18	.78	.16^†^	.34***	.16	.07	.20*		
7. Physics identification baseline	4.40	1.40	.30***	.04	.37***	.36**	.13	.01	
8. Physics identification post	4.22	1.48	.43***	.16*	.39***	.38***	.26***	.11	.71***

All correlations are one-tailed; *p < .05, **p < .01, ***p < .001, ^†^*p* < .10; *N* varies between 160 and 100 depending on the variables in question

### Gender differences in belonging

It was predicted that women would report less belonging in the courses than men (hypothesis 1), but no significant gender differences would emerge for belonging to the university. Results from two sets of independent samples *t* tests supported this hypothesis. Women reported marginally lower course belonging (*M* = 5.13, SD = 1.47) than men (*M* = 5.55, SD = 1.24), *t*(67.42) = − 1.69, *p* = .09, 95% CI [−.92, .076]. No significant gender differences emerged for university belonging, *n.s.* Results for hypothesis 1 approached marginal significance with a Mann-Whitney *U* non-parametric *t* test (*p* = .10).

### Gender differences in physics identification

Hypothesis 2 predicted that men would evidence higher physics identification at baseline and at the end of the term than women. Results from two independent samples *t* tests provided support for the idea that: (a) men reported significantly higher physics identification at baseline (*M* = 4.59, SD = 1.30) than women at baseline (*M* = 3.89, SD = 1.54), *t*(59.87) = − 2.56, *p* = .01, 95% CI [− 1.27, −.15]; and (b) men reported significantly higher physics identification at the end of the term (*M* = 4.39, SD = 1.38) than women at the end of the term (*M* = 3.77, SD = 1.65), *t*(66.99) = − 2.21, *p* = .03, 95% CI [− 1.17, −.06]. Results were replicated with Mann-Whitney *U* non-parametric *t* tests.

### Gender differences in academic achievement

Hypotheses 3a and 3b posited that men would score higher on knowledge test (FCI scores) in physics and earn higher grades than women. Results from two independent samples *t* tests only yielded significant gender differences for FCI scores. Specifically, men (*M* = 16.71, SD = 6.35) scored significantly better on the FCI at the end of the term than women (*M* = 13.70, SD = 6.04), *t*(48.60) = − 2.18, *p* = .03, 95% CI [− 5.78, −.24]. Following the suggestion of one of the manuscript reviewers, we conducted some additional analyses to shed additional light on gender differences on the FCI. Results from an independent sample *t* test examining gender differences on FCI at the beginning of the term showed that men (*M* = 13.52, SD = 5.96) also scored significantly higher on the FCI at the *beginning* of the term than women (*M* = 9.81, SD = 5.60), *t*(49.29) = − 2.89, *p* = .01, 95% CI [− 6.28, − 1.13]. However, results showed no gender differences on the difference score of FCI (post FCI-baseline FCI), *n.s*. This suggests that men began the physics course with significantly more physics knowledge than women. However, over the course of the term, males and females made comparable gains in physics knowledge, but women still significantly lagged behind men in physics knowledge by the end of the course. In fact, women’s mean FCI score at the end of the term (*M* = 13.70) was comparable to men’s FCI at the beginning of the term (*M* = 13.52).

### The relationship of physics identification and academic achievement

Hypotheses 4–5 predicted that controlling for pre-term physics knowledge (FCI scores), higher physics identification at the end of the term would predict higher scores on a physics knowledge test (FCI scores) (hypothesis 4a) and higher physics course grades (hypothesis 4b**)**. Additionally, controlling for pre-term physics knowledge (FCI scores), higher post physics knowledge scores (hypothesis 5a) and physics course grades (hypothesis 5b) would be related to more physics identification at the end of the term. A series of four step-wise hierarchical regressions were conducted to test these hypotheses. In all the step-wise hierarchical regressions, the first step (model A) involved regressing pre-term FCI scores on the dependent variable, which consisted of either post FCI scores (hypothesis 4a) or physics course grades (hypothesis 4b). The last step (model B) added either physics identification (hypotheses 4a–b) or post FCI scores (5a–b) to the regression equation. Results showed significant effects only for the regression analyses involving physics course grades (hypothesis 4b and hypothesis 5b), but not those pertaining to FCI scores (hypotheses 4a and 5a). Tables 2 and 3 show the results for model A and model B for the two hierarchical regression analyses involving physics course grades. Specifically, results from one regression analysis showed that controlling for baseline physics knowledge, higher physics identification significantly predicted higher course grades. Results from the other regression analysis showed that controlling for baseline physics knowledge, students who earned higher course grades tended to identify more with physics. Overall, the pattern of results from these two regression analyses highlights a bidirectional relationship between physics identification and grades (see Tables 4 and 5).

**Table 4:** Regression models for physics identification on course grades

	Model	
	A	B
FCI baseline	06	.05
	.38*	.31*
	(.02)	(.02)
Physics identification		.14
		.21*
		(.06)
*R* ^2^	.15	.18
*F*	17.31	11.22
*p* > *F*	.0001	.0001
*N*	99	99

*p < .05. Values in each cell are unstandardized regression coefficients, standardized regression coefficients, and standard errors

**Table 5:** Regression models for physics course grades on physics identification

	Model	
	A	B
FCI baseline	.04	.02
	.15*	.09
	(.02)	(.02)
Physics identification baseline	.74	.74
	.69*	.6*
	(.08)	(.08)
Physics course grades		.27
		.17*
		(.120)
*R* ^2^	.57	.59
*F*	57.65	41.97
*p* > *F*	.0001	.0001
*N*	89	89

*p < .05. Values in each cell are unstandardized regression coefficients, standardized regression coefficients, and standard errors

### How physics identification and gender are related to flourishing

**Fig. 2:**
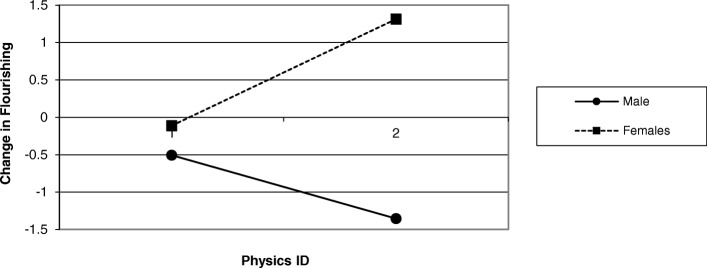
Changes in flourishing over the course of the term based on physics identification for males and females. Positive numbers for flourishing indicate positive change for flourishing. Negative numbers indicate a negative change for flourishing

Hypothesis 6 predicted that all high identifiers would report gains in flourishing over the course of the quarter than low identifiers, particularly for women. This hypothesis was tested with a step-wise regression. We began the analyses by regressing a dummy-coded gender variable (female = − 1, male = 1) on the flourishing change variable (difference score of baseline and post course flourishing) outcome (model A). In the next step, we added baseline physics identification into the regression equation (model B). In the final step, we examined whether physics identification was moderated by gender by adding the product term of gender and physical identification (model C) into the regression equation. Table 6 shows the results for all three of the models. Results suggested that model C provided the best overall fit for flourishing change scores and yielded a significant interaction (see Fig. 2). Follow-up simple slope analyses for the association of physics identification on flourishing change were tested for males and females separately. In line with expectations, results from simple slope tests for women showed that higher physics identification was associated with positive gains in flourishing over time (β = .24, SE = .08, *t*(135) = 2.68, *p* = 0.008). On the other hand, results from simple slope analyses for men showed that higher physics identification was (surprisingly) associated with *less* flourishing over time (β = −.14, SE = .07, *t*(135) = − 2.17, *p* = 0.03).

**Table 6:** Regression models for gender and physics identification on flourishing change

	Model		
	A	B	C
Gender	.03	.03	−.009
	.04	.04	−.01
	(.07)	(.07)	(.08)
Physics identification		.002	.05
		.01	.09
		(.05)	(.05)
Gender * physics identification			−.21*
			−.27
			(.07)
*R* ^2^	.01	.01	.07
*F*	.17	.09	3.18*
*p* > *F*	.67	.92	.003
*N*	138	138	138

*p < .01. Values in each cell are unstandardized regression coefficients, standardized regression coefficients, and standard errors. Also note that flourishing change refers to the difference score of baseline and end of the term flourishing

## Discussion

The goal of the current study was to examine the longitudinal influence of STEM identity for women and men in an introductory physics university course for STEM majors from a positive education perspective. Various insights emerged from this research with important implications for STEM education. First, results showed that the social psychological experiences of women in physics classrooms may be different from those of men. Women STEM majors reported marginally less course belonging after taking an introductory physics course. Women also identified less with the field of physics both at beginning and at the end of the term than men. Of note, there were no gender differences reported in belonging at the university level, which suggests that the university environment may be relatively inclusive of women but that the situation inside the classroom is different. This underlines the need for additional research efforts to further examine classroom social environments and shed light on any aspects of the social environment within physics classrooms that may be contributing to gender differences in belonging and identification. Classroom climate may be a critical component of helping retain women in STEM, particularly for universities that have a predominantly commuter student population such as the current sample, where only 9% live on campus.

Second, the current study showed that men scored higher on the FCI at the beginning and at the end of the term than women, but no significant gender differences emerged in FCI difference scores in FCI or for course grade. Gender differences on the FCI have consistently emerged in previous research (e.g., Docktor and Heller, 2008). McCullough (2011) attributes the persistent gender disparity in FCI scores to the stereotypically male contexts of FCI questions. For example, FCI items pertain to hockey, rockets, and cannonballs, which may bias the test in favor of men. However, efforts to change the context of the questions to match more stereotypical female contexts have not been fruitful in increasing FCI scores for women (McCullough, 2011). Clearly, this points to the need for more research on factors that may explain these persistent gender disparities on the FCI, despite the fact men and women seem to make comparable gains in FCI during the course. Does this pattern of gender disparities persist beyond introductory physics? This is an important question for future research because persistent knowledge disparities underline that women may be at disadvantage compared to men. It is not clear what type of effect a persistent knowledge disparity may have on women’s overall performance in their major, their well-being, or their career choices. Besides the stereotypically male contexts of the FCI questions, another possibility that may be considered for future research is that gender disparities in FCI scores may be attributable to social identity threat (Steele et al. 2002; Steele and Aronson, 1995). As such, perhaps changing the social environment in ways that reduce social identity threat may affect FCI scores.

Third, results revealed that both male and female STEM majors who identified with the field of physics received higher grades in the introductory physics course. These findings corroborate research findings from Europe and Australia underlining the importance of discipline identification for achievement outcomes (Bliuc et al. 2011; Smyth et al. 2013). They suggest that future efforts should be geared towards developing ways to encourage undergraduates to forge a discipline-based social identity as a potential means of increasing academic achievement and discipline related knowledge. In line with the findings of Platow and colleagues (2013), results also provided support for the idea that earning higher grades predicts higher physics identification. As such, performing well in the course affects the way that students viewed themselves and how much they believed they shared commonalities with the discipline. This implies that helping students forge a discipline-related identity may be accomplished via targeting their academic performance (operationalized as course grade). Overall, the current study points to the idea that the relationship between physics identification and grades is bidirectional. That is, better academic performance is associated with higher physics identification and in turn, physics identification is related to better academic performance. In this way, the identification-performance link may potentially be like a feedback loop that is recursive and cyclical (see Sherman et al. 2013).

Fourth, the current study tested the key idea that women who identify with the field of physics will show positive flourishing change over the course of the term. Results supported this hypothesis. This finding underlines the idea that STEM identification is not only connected with academic achievement but also serves as an important source of flourishing for women. This may be the case because of the benefits—both mental and physical—accrued from identification include having the needs satisfied (Greenaway et al. 2016) of the *social self*. That is, identifying with STEM may have a “protective effect”^2^ in the social arena, that is, it helps women to make gains in flourishing over the term despite facing the task of navigating a social environment where they are numerical minorities in the classroom.

This same pattern of results does not hold up for men. Higher physics identification for men was related to significantly *less* flourishing over the course of the term. Why is higher physics identification was associated with decreased flourishing for men over the course of the term, but it seems to have a protective effect for women? This is a potentially important finding that warrants replication in other samples and merits further research.

One potential explanation for these results is that the relationship of STEM identification, gender, and well-being is much more complex than we originally conceived. It is possible that the *gender of the professor* of the course may differentially influence students’ experiences in the course for high physics-identifying male and female students. High-identifying students may look to professors not only to learn about course content but also to learn about the prototypes of the field. As such, professors may act as role models and leaders (Hogg, 2001) inside the classroom who may advertently or inadvertently convey important information about the prototypes of their academic fields. In particular, professors’ cross-cutting dimensions of identity are readily on display to students and project the typical features of those who succeed in the field.

Given that the field of physics tends to be male-dominated, the physics prototype may intersect with gender (e.g., physicists tend to be male professors) and high identifiers may also take into account the gender of the professor in their overall perception of physics prototypes. In a male-dominated field such as physics, male professors may be perceived as more closely aligned with the physicist prototype at the beginning of the term than female professors. Since prototypes are context-specific and malleable (Seyranian, 2014), over the course of the term, the professor may convey important information about the field’s prototypes including gender. High-identifying students may be eager to “fit in” and be particularly attuned to whether they match these prototypes. A high student-prototype match may be related to more flourishing and a low match may negatively affect flourishing for students. For instance, women enrolled in a course with a female professor may show positive gains in flourishing because of (gender) prototype congruency. Pearson correlations (one-tailed) from the current study indicated that women enrolled in a physics course with a female instructor showed positive gains in flourishing at the end of the term (*r* = .34, *p* < .10, *n* = 27). Due to the small sample size, these results should be interpreted with caution and replicated in other samples, but they do suggest that exposure to a female prototype of a physicist may have provided female students with hopes of success for minorities “like me,” which may positively influence women’s changes in flourishing levels. In other words, the presence of a female professor may help to buffer some of the negative experiences of being in a male-dominated field for women. This idea is supported by previous research. Undergraduate women who take math and science courses with women professors are more likely to report interest and anticipate success in STEM fields, and to more strongly associate *women* with *leadership* on implicit measures (Dasgupta and Asgari 2004; Stout et al. 2011; for review, see Dasgupta, 2013). These effects are especially pronounced when undergraduate women perceive themselves to be similar to successful women leaders by, for example, being associated with the same university (Asgari et al. 2012).

Male students with a female professor may display negative change in flourishing over the course of the term because exposure to a female professor reduces the perceived strength of the male physics prototype and creates the impression that the prototype of the field of physics is changing as women gain prominence. Pearson correlations (one-tailed) from the current study support this idea. Male students enrolled in the physics course with a female professor showed negative changes in flourishing at the end of the term (*r* = −.16, *p* < .10, *n* = 74), but the same pattern did not emerge for males with a male professor (*r* = .20, *p* > .10, *n* = 28). This suggests that changes in prototypes may negatively affect members of the dominant group. Prislin and colleagues’ research consistently shows that in the aftermath of social change, there is considerable tension between majority and minority factions, and previous majority group members respond unfavorably to a relegated status (e.g., Prislin and Christensen, 2005; Prislin et al. 2000). These speculations await rigorous empirical verification in future research.

### Future research on STEM identity

The results of the current research further outline a number of areas for future research on STEM identity. This study underlines the key idea that STEM identity plays an important role in academic achievement and flourishing and that these relationships are complex and tied to intersecting identities such as gender and social environmental cues (see also Mavor et al. 2014). Research further investigating these relationships is warranted to provide a more thorough scientific understanding of the role of STEM identity in student academic success and flourishing.

Another important avenue of future research is to further investigate the malleability of STEM identities and prototypes in higher education. Prototypes are context specific and subject to change (Seyranian, 2013, 2014, 2017). Therefore, it is possible for efforts geared at creating more inclusive STEM prototypes that include women to succeed.

Lastly, careful attention must be paid to students’ physical and social learning environments, which may subtly or not so subtly send cues about who belongs and succeeds in STEM fields (Murphy and Walton, 2013). Several studies find that computer science classrooms filled with cues associated with science fiction, such as video games, significantly depress women’s interest, predicted success, and belonging in computer science, in contrast to “neutral” rooms lacking social-identity markers (Cheryan et al. 2009; Cheryan et al. 2011; Master et al. 2016). A central question for STEM researchers, educators, and practitioners remains: what messages does the diversity of our faculty and the contents of our classrooms, offices, exams, and syllabi send about *who* belongs in our field and *what* it means to be a member of the field?

### Limitations

The current study is limited in various ways. The sample size precluded a full test of some of the hypothesized relationships in the current study, and it will be important to test complex hypotheses in a larger sample. Moreover, as is common with longitudinal studies, there was some attrition in the current study that further reduced sample size. Introductory STEM courses are critical transition courses for STEM majors and reflect important psychological processes for STEM identity. Physics is a particularly important discipline in which to explore gender and identity, but physics samples tend to be small, with fewer students pursuing majors in the physical sciences. Indeed, the current study had just one student in the sample who intended to major in physics, with the remaining students intending to major in a STEM field (e.g., engineering). It is possible that STEM identity processes may differ for those who intend to major in a given field. As such, the results of the current study should also be replicated in samples of physics majors.

## Conclusions

The current study investigated responses to the social climate of physics classrooms by examining gender differences in course belonging. Next, the study examined STEM identity and gender disparities on achievement and changes in flourishing in an introductory physics course for STEM majors. Results highlighted that women report less belonging in physics courses, less physics identification (baseline and post) and less physics knowledge at the end of the term. It seems that the experience of women in physics classrooms is different from those of men. Another insight that emerged from the current study is that a strong STEM identity at the beginning of the term in a discipline is associated with stronger academic performance and positive gains in flourishing in at the end of the term for women. As such, the current research highlights that interventions that strengthen STEM identification for women may signal one promising approach to reduce gender disparities. Additionally, we outline a number of areas for future research inquiry, including more closely examining the social identity complexity of STEM identities (for example, the intersection of STEM and gender), studying the malleability of STEM-based prototypes in education, investigating how to develop education interventions that increase STEM identification and alter the content of identity to boost students’ feelings of belonging, academic performance, and positive flourishing. With further work on STEM identity in undergraduate settings from a positive education perspective that includes a focus on both academic achievement and well-being, the STEM experience can blossom into an inclusive experience for *all* types of students from *all* walks of life to ensure success for all.

## Appendix

Flourishing Scale (adapted from Diener et al. 2010).

Please indicate how true each statement is for you:

I lead a purposeful and meaningful life.

My social relationships are supportive and rewarding.

I am engaged and interested in my daily activities.

I actively contribute to the happiness and well-being of others.

I am competent and capable in the activities that are important.

I am a good person and live a good life.

I am optimistic about my future.

People respect me.

## Footnotes

1 We initially included gender in all analyses to examine whether gender moderates the relationship between identification and academic achievement (post-term FCI and course grades) while controlling for pre-term FCI scores. No significant effects were found for gender as a moderator in any of the regression equations. As such, we elected to exclude gender from these analyses.

2 We would like to acknowledge one of our reviewers who framed the flourishing effect for women in terms of the “protective” effect of social identification.

## References

[CR1] Abramzon N, Benson P, Bertschinger E, Blessing S, Cochran GL, Cox A, Yennello S (2015). Women in physics in the United States: recruitment and retention. AIP Conference Proceedings.

[CR2] Adler A, White MA, Slemp GR, Murray AS (2017). Well-being and academic achievement: Towards a new evidence-based educational paradigm. Future directions in well-being: Education, organizations and policy.

[CR3] American College Health Association – National College Health Assessment. 2015. ACHA NCHA survey. Retrieved from https://www.acha.org/NCHA/About_ACHA_NCHA/Survey/NCHA/About/Survey.aspx?hkey=7e9f6752-2b47-4671-8ce7-ba7a529c9934.

[CR4] American Physics Society (2015). Fraction of Bachelor’s Degrees in STEM disciplines earned by women.

[CR5] American Psychology Association (2018). Campus Mental Health.

[CR6] Asgari Shaki, Dasgupta Nilanjana, Stout Jane G. (2011). When Do Counterstereotypic Ingroup Members Inspire Versus Deflate? The Effect of Successful Professional Women on Young Women’s Leadership Self-Concept. Personality and Social Psychology Bulletin.

[CR7] Barbercheck, M. (2001). Science, sex, and stereotypical images in scientific advertising. In M. Wyer, M. Barbercheck, D. Giesman, H.Ö. Öztürk, & M. Wayne (Eds.), Women, science, and technology: A reader in feminist science studies (2nd ed., pp. 118–132). New York: Cambridge University Press.

[CR8] Baumeister RF, Leary MR (1995). The need to belong: Desire for interpersonal attachments as a fundamental human motivation. Psychol Bull.

[CR9] Bizumic B, Reynolds KJ, Turner JC, Bromhead D, Subasic E (2009). The role of the group in individual functioning: School identification and the psychological well-being of staff and students. Applied Psychology: An International Review.

[CR10] Bliuc A, Ellis RA, Goodyear P, Hendres DM (2011). Understanding student learning in context: Relationships between university students’ social identity, approaches to learning, and academic performance. Eur J Psychol Educ.

[CR11] Butler J, Kern ML (2016). The PERMA-profiler: A brief multidimensional measure of flourishing. Int J Wellbeing.

[CR12] Cameron J (1999). Social identity and the pursuit of possible selves: Implications for the psychological well-being of university students. Group Dyn Theory Res Pract.

[CR13] Carli LL, Alawa L, Lee Y, Zhao B, Kim E (2016). Stereotypes about gender and science: Women ≠ scientists. Psychol Women Q.

[CR14] Chemers MM, Zurbriggen EL, Syed M, Goza BK, Bearman S (2011). The role of efficacy and identity in science career commitment among underrepresented minority students. J Soc Issues.

[CR15] Cheryan S, Meltzoff AN, Kim S (2011). Classrooms matter: The design of virtual classrooms influences gender disparities in computer science classes. Com Educ.

[CR16] Cheryan S, Plaut VC, Davies P, Steele CM (2009). Ambient belonging: How stereotypical cues impact gender participation in computer science. J Personal Soc Psychol.

[CR17] Cruwys T, Dingle GA, Haslam C, Haslam SA, Jetten J, Morton T (2013). Social group memberships protect against future depression, alleviate depression symptoms and prevent depression relapse. Soc Sci Med.

[CR18] Dasgupta N, Devine P, Plant A (2013). Implicit attitudes and beliefs adapt to situations: A decade of research on the malleability of implicit prejudice, stereotypes, and the self-concept. Advances in experimental social psychology volume 47.

[CR19] Dasgupta N, Asgari S (2004). Seeing is believing: Exposure to counterstereotypic women leaders and its effect on the malleability of automatic gender stereotyping. J Exp Soc Psychol.

[CR20] Diener E, Wirtz D, Tov W, Kim-Prieto C, Choi D, Oishi S, Biwas-Diener R (2010). New well-being measures: Short scales to assess flourishing and positive and negative feelings. Soc Indic Res.

[CR21] Docktor J, Heller K (2008). Gender differences in both force concept inventory and introductory physics performance. 2008 physics education research conference. AIP conference proceedings, 1064.

[CR22] Ellemers N, Spears R, Doosje B (2002). Self and social identity. Ann Rev Psychol.

[CR23] Fredrickson BL, Losada MF (2005). Positive affect and the complex dynamics of human flourishing. Am Psychol.

[CR24] Funk C, Parker K (2018). Women in STEM see more gender disparities at work, especially those in computer jobs, majority-male workplaces. Pew Research Center: Social and Demographic Trends.

[CR25] Greenaway KR, Cruwys T, Haslam SA, Jetten J (2016). Social identities promote well- being because they satisfy global psychological needs. Eur J Soc Psychol.

[CR26] Grobecker PA (2016). A sense of belonging and perceived stress among baccalaureate nursing students in clinical placement. Nur Educ Today.

[CR27] Hestenes D, Wells M, Swackhamer G (1992). Force concept inventory. Phys Teach.

[CR28] Hogg MA (2001). A social identity theory of leadership. Pers Soc Psychol Rev.

[CR29] Hogg Michael A. (2007). Uncertainty–Identity Theory. Advances in Experimental Social Psychology.

[CR30] Hogg MA, Abrams D, Hogg MA, Abrams D (2001). Intergroup relations: An overview. Intergroup relations.

[CR31] Huppert FA, So TTC (2013). Flourishing across Europe: Application of a new conceptual framework for defining well-being. Soc Indic Res.

[CR32] Ivie R, Guo S (2006). Women physicists speak again. American Institute of Physics report.

[CR33] Ivie R, White S (2015). Is there a land of equality for physicists? Results from the global survey of physicists. La Physique Au Canada.

[CR34] Ivie R, White S, Garrett A, Anderson G (2013). Representation of women continues to grow.

[CR35] Kim Ann Y., Sinatra Gale M., Seyranian Viviane (2018). Developing a STEM Identity Among Young Women: A Social Identity Perspective. Review of Educational Research.

[CR36] Master A, Cheryan S, Meltzoff AN (2016). Computing whether she belongs: Stereotypes undermine girls’ interest and sense of belonging in computer science. J Educ Psychol.

[CR37] Mavor KI, McNeill KG, Anderson K, Kerr A, O’Reilly E, Platow MJ (2014). Beyond prevalence to process: The role of self and identity in medical student well-being. Med Educ.

[CR38] McCullough, L. (2011). Gender differences in student responses to physics conceptual questions based on question context. ASQ Advancing the STEM Agenda in Education Session 2-3, University of Wisconsin-Stout, July 19-20. Retrieved from http://asq.org/edu/2011/06/continuous-improvement/gender-differences-in-student-responses-to-physics-conceptual-questions-based-on-question-content.pdf.

[CR39] Murphy MC, Steele CM, Gross JJ (2007). How situational cues affect women in math, science, and engineering settings. Psychol Sci.

[CR40] Murphy MC, Walton GM, Stangor C, Crandall C (2013). From prejudiced people to prejudiced places: A social-contextual approach to prejudice. Frontiers in social psychology series: Stereotyping and prejudice.

[CR41] Murphy MC, Zirkel S (2015). Race and belonging in school: How anticipated and experienced belonging affect choice, persistence, and performance. Teach Coll Rec.

[CR42] Nakamura, J., & Csikszentmihalyi, M. (2009). Flow theory and research. In S.J. Lopez and C.R. Snyder (Eds.), The Oxford Handbook of Positive Psychology (2nd Ed., pp. 195–206). New York, NY: Oxford University Press, Inc.

[CR43] National Science Foundation (2015). Women, minorities, and persons with disabilities in science and engineering. Retrieved from http://www.nsf.gov/statistics/2015/nsf15311/digest/nsf15311-digest.pdf.

[CR44] Platow MJ, Mavor KI, Grace DM (2013). On the role of discipline-related self-concept in deep and surface approaches to learning among university students. Instr Sci.

[CR45] Prislin R, Christensen PN (2005). Social change in the aftermath of successful minority influence. Eur Rev Soc Psychol.

[CR46] Prislin R, Limbert WM, Bauer E (2000). From majority to minority and vice versa: The asymmetrical effects of losing and gaining majority position within a group. J Pers Soc Psychol.

[CR47] Rainey, K., Dancy, M., Mickelson, R., Stearns, E., & Moller, S. (2018). Race and gender differences in how sense of belonging influences decisions to major in STEM. *Int J STEM Educ, 5*. 10.1186/s40594-018-0115-6. 10.1186/s40594-018-0115-6 PMC6310405 30631700

[CR48] Robnett RD (2012). The role of peer support for girls and women in the STEM pipeline: Implications for identity and anticipated retention. International Journal of Gender, Science and Technology.

[CR49] Robnett RD, Thoman SE (2017). STEM success expectancies and achievement among women in STEM majors. J Appl Dev Psychol.

[CR50] Schreiner, L. A., Hulme, E., Hetzel, R., & Lopez, S. J. (2009). Positive psychology on campus. In. S. J. Lopez and C. R. Snyder (Eds.), *The Oxford Handbook of Positive Psychology* (2nd Ed., pp. 569–578). New York: Oxford University Press, Inc.

[CR51] Seligman M, Ernst R, Gillham K, Reivich K, Linkins M (2009). Positive education: Positive psychology and classroom interventions. Oxf Rev Educ.

[CR52] Seligman MEP (2011). Flourish: A visionary new understanding of happiness and well- being.

[CR53] Seymour, E., & Hewitt, N. M. (1997). *Talking about leaving: Why undergraduates leave the sciences*. Boulder: Westview Press.

[CR54] Seyranian V, Riggio RE, Tan SJ (2013). Social identity framing: A strategy of social influence for social change. Leader interpersonal and influence skills: The soft skills of leadership.

[CR55] Seyranian V (2014). Social identity framing communication strategies for mobilizing social change. Leadersh Q.

[CR56] Seyranian V (2017). Public interest communication: A social psychological perspective. Journal of Public Interest Communications.

[CR57] Sherman David K., Hartson Kimberly A., Binning Kevin R., Purdie-Vaughns Valerie, Garcia Julio, Taborsky-Barba Suzanne, Tomassetti Sarah, Nussbaum A. David, Cohen Geoffrey L. (2013). Deflecting the trajectory and changing the narrative: How self-affirmation affects academic performance and motivation under identity threat. Journal of Personality and Social Psychology.

[CR58] Smyth L, Mavor KI, Platow MJ, Grace DM, Reynolds KJ (2013). Discipline social identification, study norms and learning approach in university students. Educ Psychol.

[CR59] Steele CM, Aronson J (1995). Stereotype threat and the intellectual test performance of African Americans. J Pers Soc Psychol.

[CR60] Steele J, James JB, Barnett RC (2002). Learning in a man’s world: Examining the perceptions of undergraduate women in male-dominated academic areas. Psychol Women Q.

[CR61] Stout JG, Dasgupta N, Hunsinger M, McManus MA (2011). STEMing the tide: Using ingroup experts to inoculate women’s self-concept in science, technology, engineering, and mathematics (STEM). J Pers Soc Psychol.

[CR62] Tajfel, H. & Turner, J. C. (1986). The social identity theory of intergroup behavior. In S. Worchel & W. G. Austin (Eds.), *Psychology of Intergroup Relations* (2nd ed., pp. 7–24). Chicago: Nelson-Hall.

[CR63] Townsend SSM, Major B, Gangi CE, Mendes WB (2011). From ‘in the air’ to ‘under the skin’: Cortisol responses to social identity threat. Pers Soc Psychol Bull.

[CR64] Traxler, A., Henderson, R., Stewart, J., Stewart, G., Papak, A., & Lindell, R. (2018). Gender fairness within the Force Concept Inventory. *Phys Rev Phys Educ Res, 14*. 10.1103/PhysRevPhysEducRes.14.010103.

[CR65] Turner JC (1991). Social influence.

[CR66] van Laar C, Derks B, Ellemers N, Bleeker D (2010). Valuing social identity: Consequences for motivation and performance in low-status groups. J Soc Issues.

[CR67] Walton GM, Cohen GL (2007). A question of belonging: Race, social fit, and. achievement. J Pers Soc Psychol.

